# 5-HT3 Signaling Alters Development of Sacral Neural Crest Derivatives That Innervate the Lower Urinary Tract

**DOI:** 10.3390/ijms22136838

**Published:** 2021-06-25

**Authors:** K. Elaine Ritter, Dennis P. Buehler, Stephanie B. Asher, Karen K. Deal, Shilin Zhao, Yan Guo, E Michelle Southard-Smith

**Affiliations:** 1 Division of Genetic Medicine, Department of Medicine, Vanderbilt University Medical Center, Nashville, TN 37232, USA; ritterka@med.umich.edu (K.E.R.); dennis.p.buehler@vumc.org (D.P.B.); stephanie.asher@pennmedicine.upenn.edu (S.B.A.); karen.k.deal@vumc.org (K.K.D.); 2 Department of Biostatistics, Vanderbilt University Medical Center, Nashville, TN 37232, USA; shilin.zhao.1@vumc.org (S.Z.); yaguo@salud.unm.edu (Y.G.)

**Keywords:** pelvic ganglia, bladder, autonomic nervous system, serotonin, *Sox10*, *Htr3a*

## Abstract

The autonomic nervous system derives from the neural crest (NC) and supplies motor innervation to the smooth muscle of visceral organs, including the lower urinary tract (LUT). During fetal development, sacral NC cells colonize the urogenital sinus to form pelvic ganglia (PG) flanking the bladder neck. The coordinated activity of PG neurons is required for normal urination; however, little is known about the development of PG neuronal diversity. To discover candidate genes involved in PG neurogenesis, the transcriptome profiling of sacral NC and developing PG was performed, and we identified the enrichment of the type 3 serotonin receptor (5-HT3, encoded by *Htr3a* and *Htr3b*). We determined that *Htr3a* is one of the first serotonin receptor genes that is up-regulated in sacral NC progenitors and is maintained in differentiating PG neurons. In vitro cultures showed that the disruption of 5-HT3 signaling alters the differentiation outcomes of sacral NC cells, while the stimulation of 5-HT3 in explanted fetal pelvic ganglia severely diminished neurite arbor outgrowth. Overall, this study provides a valuable resource for the analysis of signaling pathways in PG development, identifies 5-HT3 as a novel regulator of NC lineage diversification and neuronal maturation in the peripheral nervous system, and indicates that the perturbation of 5-HT3 signaling in gestation has the potential to alter bladder function later in life.

## 1. Introduction

Dysfunction of the urinary bladder can present as urinary incontinence, neurogenic bladder, or urinary retention. These disorders afflict millions of people worldwide and dramatically impact quality of life [1,2]. In many cases, bladder dysfunction is caused by damage to the nerves that supply the LUT. The cell bodies of the autonomic neurons that innervate the bladder are situated some distance away from the bladder itself, unlike many other autonomic neurons that are in close proximity to their target organs. Thus, the processes of these pelvic neurons are prone to damage during pelvic surgical procedures or childbirth. In addition, pelvic autonomic neurons have limited capacity to regenerate their processes in vivo following axotomy, in contrast to sensory neurons that regenerate their axons rapidly [3]. As a result, there is growing interest in understanding the developmental processes that govern the formation and maturation of LUT innervation as a means to devise novel strategies to restore bladder function [4].

Normal bladder function relies on autonomic neural inputs to coordinate the relaxation and contraction of the bladder and urethral sphincter. In mice, autonomic innervation of the LUT is provided by the major pelvic ganglia, which consist of thousands of sympathetic and parasympathetic neurons mixed together in paired ganglia that lie close to the uterine cervix in females and flank the prostate in males. Initial efforts to discern factors that regulate pelvic ganglia neurogenesis relied on a genome-wide screen of transcription factors via high-throughput whole-mount in situ hybridization of fetal mouse urinary tracts [5]. Key transcription factors, including those that have known roles in NC development and autonomic neurogenesis, such as *Phox2b*, *Gata2*, and *Hand2*, were identified. However, relationships between these factors and the expression and function of downstream signaling pathways in pelvic ganglia development have remained elusive.

Relatively little is known about the signaling factors that impact early development and neurogenesis within the pelvic ganglia despite the great need to identify molecules that could be used to promote the repair of damaged pelvic innervation. To date, only a limited number of studies have examined the effects of specific molecules on fetal development and early postnatal maturation of pelvic ganglia neurons and their processes. Analyses using explant cultures of fetal mouse pelvic ganglia found that NGF, Nt-3, and members of the GDNF family exert stage-specific effects on the outgrowth of neurites from either sympathetic or parasympathetic pelvic ganglia neurons [6]. Other in vivo analyses using knockout mice elegantly parsed out the effects of disrupted neurturin signaling on the innervation of specific pelvic organs and determined that the loss of neurturin did not alter initial growth or the final density of bladder nerve terminals, but it did disrupt innervation of the vas deferens, prostate, and penis [7,8]. In contrast, much more is known about critical molecular mediators that affect the development of other aspects of the peripheral nervous system, including the enteric nervous system (reviewed by [9,10,11,12]) and sympathetic ganglia [13,14,15,16,17,18]. These advances have benefitted in part from focused efforts to profile gene expression during fetal development [19,20,21] while, to date, such information has been lacking for the pelvic ganglia.

In this study, we sought to identify novel signaling pathways that regulate neurogenesis in fetal mouse pelvic ganglia, with a particular focus on identifying factors that are amenable to pharmacologic targeting. We undertook comparative transcriptome profiling of sacral NC progenitors (NCPs) and intact fetal pelvic ganglia at developmental time points when neuronal differentiation is ongoing. We employed the *Sox10-*H2BVenus transgenic mouse line that directly labels migrating NC to illuminate both progenitor populations and the forming pelvic ganglia [22,23]. This effort identified a significant up-regulation of subunits that comprise the serotonin type 3 receptor (5-HT3) in differentiating pelvic ganglia neurons. Most notably, we observed that the serotonin receptor 3 gene, *Htr3a*, is highly expressed in sacral NCPs and is maintained in cells undergoing neuronal differentiation. The treatment of isolated *Sox10*-H2BVenus+ sacral NCPs with a specific 5-HT3 agonist revealed a previously unknown role for this receptor in modulating the differentiation potential of sacral NC lineages. Over-stimulating 5-HT3 in cultured fetal pelvic ganglia explants resulted in blunted neurite arbor outgrowth in a dose-dependent manner, which supports a role for this receptor in the morphological maturation of autonomic neurons. Altogether, our work contributes a valuable transcriptome dataset for the investigation of key molecular factors that control sacral autonomic neuronal differentiation and demonstrates a critical role for serotonin signaling via 5-HT3 in both neurogenesis and neuronal maturation in the peripheral nervous system.

## 2. Results

### 2.1. Sox10+ Neural Crest-Derived Progenitors Are Intermingled with Differentiating Neurons in the Early Pelvic Ganglia

**Figure 1:**
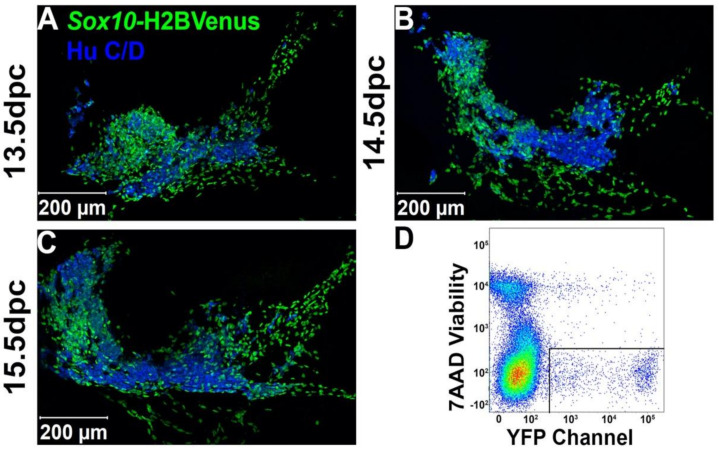
Distribution of *Sox10-*H2BVenus+ NCPs and neurons in developing fetal pelvic ganglia. *Sox10-*H2BVenus+ NC-derived cells (green) can be specifically visualized by confocal microscopy and isolated via FACs. Sagittal cryo-sections through a *Sox10-*H2BVenus transgenic mouse pelvic ganglion at (**A**) 13.5 dpc, (**B**) 14.5 dpc, and (**C**) 15.5 dpc and stained with pan-neuronal marker Hu C/D (blue cells). (**D**) FACS profile of *Sox10*-H2BVenus+ cells isolated from the urogenital sinus of 13.5 dpc transgenic mice. Viable cells that excluded 7AAD and that were Venus+ were selected as shown in the boxed gated area.

*Sox10-*H2BVenus expression labels NC-derived stem cells and is retained in mature glia [22]. However, early differentiating neurons within the anlagen of the pelvic ganglia also briefly retain residual H2BVenus fluorescence [5]. To relate the distribution of *Sox10+* cells to pelvic ganglia neurons during the development of pelvic innervation, we examined pelvic ganglia from *Sox10-*H2BVenus+ transgenic mice at fetal stages when autonomic neurogenesis is actively occurring [5]. Upon staining 13.5 dpc (days post coitus) cryo-sections with pan-neuronal marker Hu C/D, we observed Hu C/D+ neurons within the pelvic ganglia surrounded by numerous *Sox10+* cells (Figure 1A). By 14.5 dpc, the pelvic ganglia have increased in size, and the domain of Hu C/D expression is expanded (Figure 1B). Importantly, pelvic ganglia at age 15.5 dpc continue to undergo neurogenesis, as evidenced by further expansion of Hu C/D and the presence of clusters of *Sox10-*H2BVenus+ progenitor cells (Figure 1C). Not only did the robust and highly specific expression of the *Sox10-*H2BVenus transgene visualize sacral NC progenitors in situ, it also enabled the isolation of NCPs from fetal LUT via fluorescence-activated cell sorting (FACS, Figure 1D) for analysis of transcriptional profiles.

### 2.2. Expression Profiles of Differentiating Pelvic Ganglia Identify Up-Regulation of Subunits for the Serotonin Receptor 5-HT3

**Figure 2:**
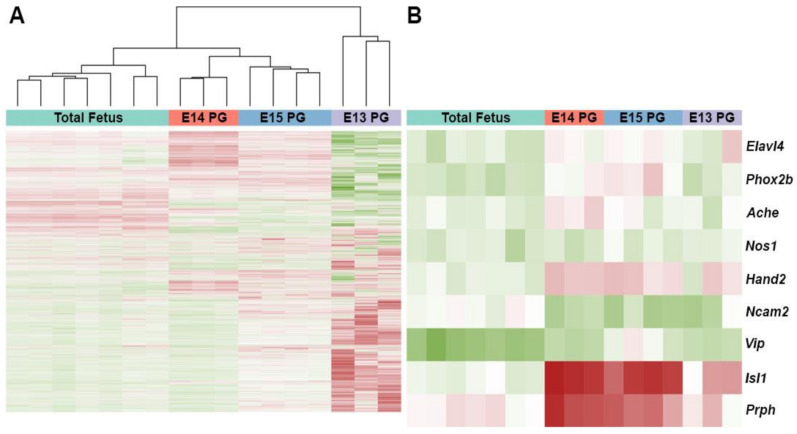
Microarray hybridization clustering of RNA isolates from fetal mouse pelvic ganglia compared to whole embryo control replicates. (**A**) Heat map displaying consistent gene expression patterns within biological replicates of control and experimental samples based on unsupervised, unbiased clustering using all genes. Each horizontal line represents expression detected by one probe set. Increased hybridization relative to control tissue is indicated by red, whereas green bars indicate decreased gene expression relative to total embryo control hybridization signals. (**B**) Heat map reflecting expression of control genes that were expected to be up-regulated in neuronal cell types across all compared samples. The graphic shown depicts clusters based on gene order.

To identify the most salient genes expressed in differentiating pelvic ganglia, we micro-dissected pelvic ganglia from *Sox10*-H2BVenus+ fetal mice at ages 13.5, 14.5, and 15.5 dpc when autonomic neurogenesis is ongoing. At 13.5 dpc, H2BVenus+ cells were harvested from dissociated pelvic ganglia by FACS to obtain transcriptional profiles for early differentiating *Sox10*+ progenitors within the pelvic ganglia. RNA was isolated and purified from intact 14.5 and 15.5 dpc pelvic ganglia so that expression profiles from differentiating neurons, which are intermingled with *Sox10*+ progenitors, were included that otherwise would have been lost if only *Sox10*-H2BVenus+ cells were collected at these stages. Then, RNA from flow-sorted *Sox10*+ progenitors and developing pelvic ganglia at 14.5 and 15.5 dpc were subjected to microarray hybridization. A complete list of Affymetric normalized hybridization intensities for each sample can be found online at the Gene Expression Omnibus (ID GSE 108943, GSM778845-GSM778854). Differentially expressed genes were identified using the LIMMA Bioconductor package (Supplementary Table S1). Biological replicates for hybridizations showed consistent tight clustering and hybridization for genes that were expected to mark differentiating neurons, including *Elavl4* (HuD), *Phox2b*, *Prph*, and *Hand2*, were up-regulated across pelvic ganglion transcriptomes (Figure 2). The top 100 most up-regulated genes were categorized as genes exhibiting the highest fold change relative to total fetus RNA labels at each stage examined (13.5 dpc Supplementary Table S2; 14.5 dpc Supplementary Table S3; 15.5 dpc Supplementary Table S4). Among these top 100 genes, we identified the significant enrichment of several genes that are characteristic of autonomic neurogenesis, including *Isl1* and *Prph* (Figure 2). We noted microarray hybridization signals for several serotonin receptor genes in RNA isolated from pelvic ganglia relative to total embryo RNA (Table 1). However, only *Htr3a*, which encodes the obligate A subunit of the pentameric 5-HT3 receptor, exhibited significant up-regulation across all three time points with the highest fold change (2.55) at 15.5 dpc (Table 1). *Htr3b*, which encodes the B subunit of 5-HT3, was also significantly up-regulated at 14.5 and 15.5 dpc with fold-change values of 1.79 and 2.15, respectively. From these transcriptional profiling experiments, we conclude that the expression of serotonin receptor 5-HT3 is significantly up-regulated in fetal pelvic ganglia during stages of autonomic neurogenesis.

### 2.3. Multiple Serotonin Receptors Are Expressed during Differentiation of Pelvic Autonomic Neurons

**Figure 3:**
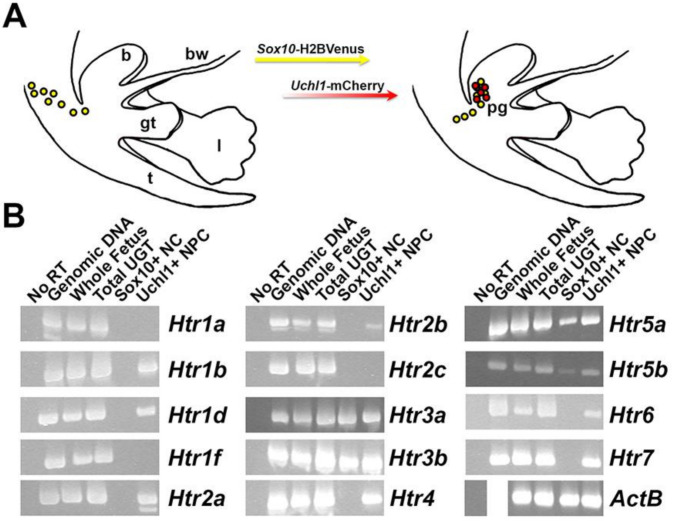
Expression of serotonin receptor genes in sacral neural crest-derived progenitors and pelvic ganglia neuronal precursors. RT-PCR conducted on cDNA derived from distinct cell populations in the lower urinary tract at 15.5 dpc reveals the expression of a subset of serotonin receptors in developing pelvic innervation. (**A**) Schematic diagram shows the distribution of cells marked by two transgenic mouse lines that were used to isolate distinct cell populations within the developing lower urinary tract. NCPs expressing *Sox10*-H2BVenus transgene (yellow) migrate into the LUT and upon neuronal differentiation up-regulate the *Uchl1*-mCherry transgene (red). cDNA was synthesized from isolated RNA that was purified from these two cell populations by FACS. (**B**) Gel electrophoresis of RT-PCR products generated by the specific amplification of 5-HT receptor genes. cDNA was synthesized from whole mouse fetus, total lower urinary tract (bladder with attached genital tubercle), *Sox10*-H2BVenus+ cells isolated by FACS, and pelvic *Uchl1*-mCherry+ neuronal progenitor RNA. Template with no reverse transcriptase added to the cDNA synthesis reaction (“No RT”) served as a negative control; genomic DNA template served as a positive control. Amplification of housekeeping gene ActB served as a positive control for the PCR reaction. (b, bladder; t, tail; gt, genital tubercle; l, limb bud; bw, body wall; pg, pelvic ganglia).

Following the detection of serotonin receptor genes in fetal pelvic ganglia by microarray hybridization, reverse-transcription (RT) PCR was performed to validate the detection of individual 5-HT receptor genes. We specifically aimed to determine whether 5-HT receptors are expressed in *Sox10*+ progenitors or differentiating neurons. To accomplish this, we synthesized cDNA using RNA isolated from two populations: flow-sorted sacral NC-derived progenitors labeled by *Sox10*-H2BVenus+ transgene expression and differentiating neurons labeled by *Uchl1*-mCherry+ transgene expression [24]. All RNA isolates were obtained from fetal mice at 15.5 dpc. We observed the expression of all serotonin receptors in cDNA generated from whole mouse fetuses, as would be expected given the wide distribution of these receptors during development. We also detected the expression of every serotonin receptor in lower urinary tract RNAs at 15.5 dpc (Figure 3). Serotonin receptor genes *Htr1b*, *Htr1d*, *Htr2a*, *Htr2b*, *Htr*4, *Htr6*, and *Htr7* are expressed in *Uchl1*+ neuronal progenitors but are not present in *Sox10*+ NCPs. Interestingly, *Htr3a*, *Htr3b*, *Htr5a*, and *Htr5b* are expressed in early neurons marked by *Uchl1*-mCherry+ transgene expression as well as in NCPs marked by *Sox10*-H2BVenus. Serotonin receptor genes *Htr1a*, *Htr1f*, and *Htr2c* are not expressed in either *Sox10*+ or *Uchl1*+ populations at 15.5 dpc. From these experiments, we conclude that several serotonin receptor family members are expressed in differentiating neurons within fetal pelvic ganglia, but only *Htr3* and *Htr5* receptor subunit genes are expressed in early NCPs at this stage.

### 2.4. Perturbing Serotonin Receptor Signaling Alters Differentiation Outcomes of Sox10+ Sacral Neural Crest Progenitors In Vitro

**Figure 4:**
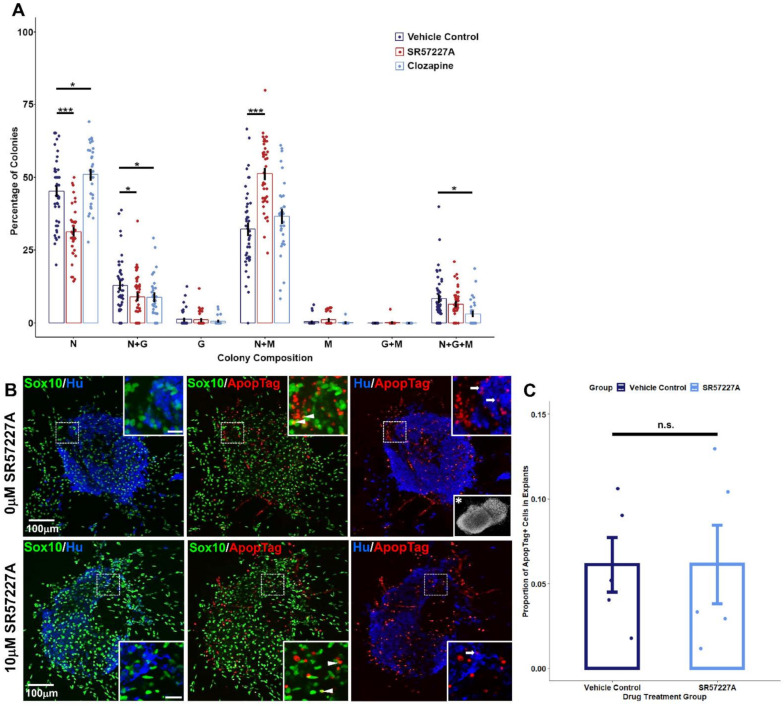
Pharmacologically perturbing serotonin signaling alters the differentiation outcomes of sacral NCPs in vitro. (**A**) *Sox10*-H2BVenus+ sacral NCPs were flow-sorted from 13.5 dpc pelvic ganglia and then grown in low-density cultures and treated with drug vehicle (negative control), SR57227A (specific 5-HT3 receptor agonist), or clozapine (mixed antagonist of several serotonin receptors). Mean ± SEM percentage of colonies expressing markers of differentiated cell types including neurons (Peripherin+, N), glia (GFAP+, G), and myofibroblast (Smooth Muscle Actin+, M) after exposure to either vehicle control, SR57227A, or clozapine are plotted. * *p* < 0.05, *** *p* < 0.001, assessed by Welch’s *t*-test. *n* = 33−45 wells per treatment condition. (**B**) Confocal images of 13.5 dpc *Sox10*-H2B-Venus (green) pelvic ganglia explants cultured in the presence or absence of the 5-HT agonist SR57227A and subjected to detection of apoptotic cells (red, ApopTag) followed by immunohistochemistry with Hu C/D antibody, a pan-neuronal marker. Occasional apoptotic glial cells (arrowheads) and neuronal cells (arrows) are indicated. The scale bar for all insets is 20 μm. Representative DAPI staining reveals the explants also contain a non-glial, non-neuronal cell population and that the majority of apoptotic events take place in this population (inset with asterisk, 120× magnification). (**C**) Quantification of proportions of ApopTag+ cells out of total cells in pelvic ganglia explants. *p* = 0.99, Welch’s *t*-test.

Given the widespread expression of serotonin receptors in developing fetal LUT tissues and specifically the sustained up-regulation of *Htr3a* across all the stages of pelvic ganglia examined, we asked what effect stimulating serotonin receptors, and 5-HT3 in particular, has on the development of sacral NCPs that give rise to pelvic innervation. To address this question, we micro-dissected LUT tissues comprised of fetal bladder, pelvic ganglia, urethra, and genital tubercle from 14.5 dpc *Sox10*-H2BVenus+ fetuses, dissociated, and flow-sorted H2BVenus+ cells directly into low-density adherent culture conditions [25,26,27,28]. Under such conditions, individual progenitors form spatially distinct colonies whose composition reflects the differentiation capacities of individual cells in vivo. Thereby, the proportions of differentiated cell types within colonies grown during exposure to serotonin-targeting drugs can be assessed by immunohistochemical staining for markers of neurons (Peripherin), glia (GFAP), and myofibroblasts (SMA) with multipotent colonies identified by the presence of all three lineages. Since we identified the expression of multiple serotonin receptors in differentiating pelvic ganglia, we opted to perturb 5-HT receptor signaling with a receptor antagonist, clozapine, which broadly targets several 5-HT receptors. The treatment of sacral NCPs with clozapine led to a modest disruption of differentiation outcomes (Figure 4, Supplementary Table S5). Clozapine treatment increased the percentage of purely neuronal colonies compared to controls (45.2 ± 1.69% vehicle control, 50.9 ± 1.7% clozapine, *p* = 0.0201, Welch’s *t*-test). Additionally, mixed neuronal/glial colonies were reduced (12.8 ± 1.38% vehicle control vs. 8.82 ± 1.28% clozapine, *p* = 0.0367, Welch’s *t*-test), as well as neuron/glia/myofibroblast colonies (8.28 ± 1.21% vehicle control vs. 3.08 ± 0.81% clozapine, *p* = 0.0005, Welch’s *t*-test).

Due to the significant up-regulation of *Htr3a* in differentiating pelvic ganglia, we also asked if over-stimulating the 5-HT3 receptor specifically would affect sacral NC differentiation outcomes. The treatment of sacral NCPs with a 5-HT3 receptor-specific agonist, SR57227A [29,30,31], resulted in a dramatic reduction in purely neuronal colonies (45.2% ± 1.69% vehicle control vs. 31.3% ± 1.33% SR57227A, *p* = 7.179 × 10^−9^, Welch’s *t*-test). Alongside the reduction in neuronal colonies, over-stimulating 5-HT3 with SR57227A also increased the percentage of mixed neuron/myofibroblast colonies compared to the vehicle control (32.2% ± 2.04% vehicle control vs. 51.2% ± 1.78%, *p* = 5.236 × 10^−10^, Welch’s *t*-test). Similar to clozapine treatment, SR57227A also led to a reduction in mixed neuronal/glial colonies compared to the vehicle control (12.8% ± 1.38% vehicle control vs. 8.93% ± 1.18% SR57227A, *p* = 0.0345, Welch’s *t*-test).

Since it is possible that the altered lineage segregation of NC could lead to aberrant cell death, we quantified proportions of apoptotic cells in our control and drug-treated pelvic ganglia explant cultures using the ApopTag assay (Figure 4B,C). We did not observe any statistically significant differences in ratios of ApopTag+ cells between vehicle control-treated and 10 µM SR57227A-treated explants (0.0361 ± 0.0161 in control vs. 0.0519 ± 0.0232 in SR57227A-treated, N = 5 explants per treatment group, *p* = 0.9933, Welch’s *t*-test).

From these experiments, we conclude that perturbing serotonin receptor signaling, particularly the 5-HT3 receptor, alters the differentiation outcomes of sacral NCPs in vitro without altering programmed cell death.

### 2.5. Over-Stimulating 5-HT3 in Pelvic Ganglia Explants Attenuates Neurite Outgrowth

**Figure 5:**
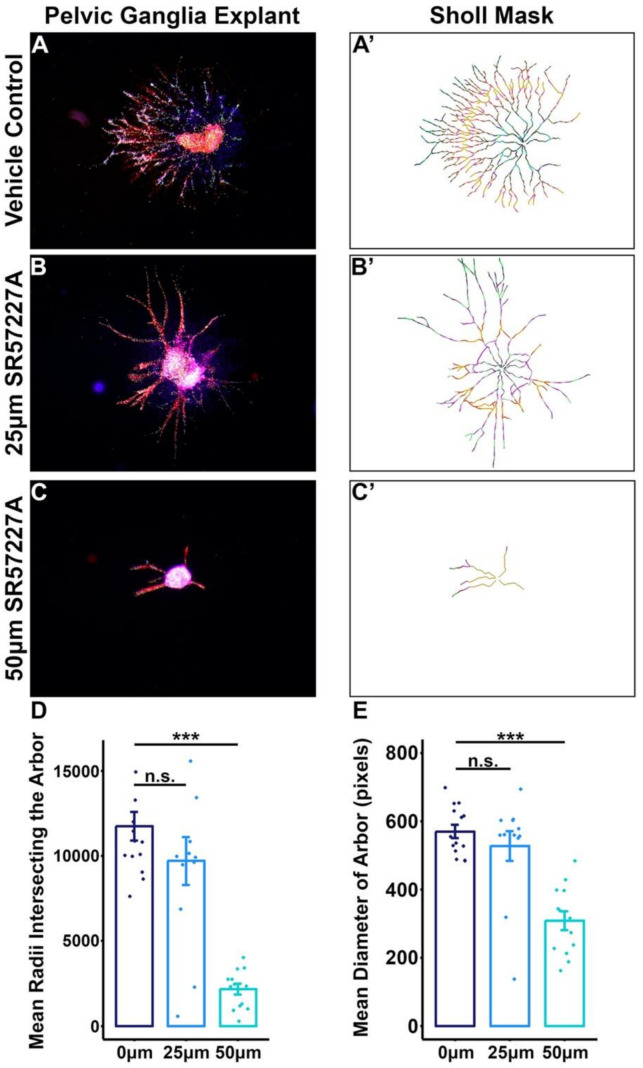
Over-stimulating 5-HT3 in cultured fetal pelvic ganglia explants diminishes neurite arbor complexity. *Sox10*-H2BVenus 13.5 dpc pelvic ganglia explants treated with 5-HT3 receptor agonist SR57227A exhibit a severe reduction in the elaboration and complexity of neuronal processes compared to vehicle control. (**A**–**C**) Fluorescent microscope images of pelvic ganglia explants treated with drug vehicle (**A**), 25 μm SR57227A (**B**), or 50 μm SR57227A (**C**). (**A’**–**C’**) Sholl masks generated for pelvic ganglia explant neuronal fiber tracings that demarcate concentric radii used to quantify neurite arbor complexity and growth. (**D**) Average number of intersections of the neurite arbor with Sholl radii plotted for each treatment group. (**E**) Average diameter of the outer-most circle drawn that encompasses the entire arbor. For both plots, black brackets are SEM. *n* = 12–14 pelvic ganglia explants per treatment group. *** = *p* < 0.001, n.s. = not statistically significant, assessed via one-way ANOVA with Tukey’s Honest Significant Difference test to correct for multiple comparisons.

Given the significant effects that over-stimulating the 5-HT3 receptor had on the developmental potential of sacral NCPs grown in culture, we sought to determine how intact pelvic ganglia explants would respond to increasing dosages of the 5-HT3 agonist SR57227A. We sub-dissected pelvic ganglia from 13.5 dpc *Sox10*-H2BVenus animals and cultured them in a collagen matrix over several days using previously described culture conditions [6]. Pelvic ganglia explants were exposed to either the drug vehicle, 25 µM, or 50 µM SR57227A. Compared to vehicle-treated control pelvic ganglia explants, SR57227A-treated explants showed diminished neurite outgrowth in a dose-dependent manner (Figure 5A–C’). Sholl analysis of neurite arbor complexity revealed a substantial decrease in the average number of radii intersecting neuritic branches (Figure 5D) (*p* = 2.319 × 10^−8^, one-way ANOVA with Tukey’s HSD post hoc test). Additionally, the average size of the arbor, as measured by the enclosing radius of the explant, was significantly reduced in SR57227A-treated cultures (Figure 5E) (*p* = 7.575 × 10^−7^, one-way ANOVA with Tukey’s HSD post-hoc test). From these experiments, we conclude that normal arborization in developing pelvic ganglia is influenced by 5-HT3 receptor signaling. 

## 3. Discussion

A variety of clinical disorders originate from the disruption of lower urinary tract innervation. Pelvic surgeries such as prostatectomy [32], hysterectomy [33,34], or colorectal cancer surgery can damage pelvic nerves and lead to subsequent incontinence. Additionally, hyper-innervation of the bladder occurs in chronic cystitis [35,36]. As a result, there is a great need to understand developmental processes that regulate pelvic innervation both for regenerative strategies to restore bladder innervation as well as for the identification of agents to modulate aberrant innervation. To date, surprisingly little has been discovered about signaling factors that function during development to govern pelvic ganglia neurogenesis and neuronal maturation. In this study, we report a transcriptome profiling dataset that consists of gene expression patterns from fetal mouse pelvic ganglia during stages of autonomic neurogenesis. This dataset offers initial access for the unbiased identification of genes that regulate the formation and development of pelvic autonomic neurons. Through bioinformatics analysis, we identified a significant up-regulation of the obligate subunit for the type 3 serotonin receptor that is encoded by *Htr3a* in *Sox10*+ sacral neural crest progenitors and maturing neurons within the pelvic ganglia anlagen. Using pharmacologic stimulation of sacral neural crest progenitors and pelvic ganglia explants, we show that serotonin signaling via 5-HT3 plays a key role in NC differentiation outcomes and neuronal maturation.

*Sox10* is an essential transcription factor for NC development, which is a well-established marker of NC-derived progenitors, and is highly expressed in sacral NC [23,37,38,39]. To readily capture NC-derived progenitors and differentiating neurons for expression profiling within developing pelvic ganglia, we took an approach that relied upon *Sox10* expression mirrored by the *Sox10*-H2BVenus transgene [22]. Since differentiating autonomic neurons intermingle with sacral neural crest-derived progenitors within the pelvic ganglia anlagen, this strategy enabled the capture of pelvic autonomic neurons in the midst of neurogenesis. Transcription profiles from micro-dissected pelvic ganglia identified an increased expression of transcription factors *Phox2b*, *Gata2/3*, and *Hand2* that is consistent with the well-established roles of these genes in orchestrating autonomic neurogenesis [40] and prior reports of their expression in fetal mouse pelvic ganglia [41,42]. Other genes that are common markers of neurogenesis including *Isl1*, *Prph*, *Elavl4* (HuD), and *Uchl1* (PGP9.5) were also observed. Among the genes that were up-regulated as pelvic ganglia differentiated were several serotonin receptors, including *Htr3a*, which is the obligatory subunit of 5-HT3.

While serotonin receptor signaling is known to play multiple critical roles in brain development [43,44,45,46], our results are the first time that signaling through 5-HT3 receptors has been implicated in autonomic neurogenesis. In the developing brain, *Htr3a* knockout models have shown that this gene impacts dendritic complexity, synaptic plasticity, and climbing fiber elimination [47,48,49,50]. Previous work in the central nervous system (CNS) has also shown that *Htr3a* influences the speed and directionality of migrating postnatal neuroblasts in the postnatal subventricular zone [51]. The notable expression of *Htr3a* in developing pelvic ganglia that we detected in this study in combination with the altered bladder function documented in *Htr3a* knockout mice [52] supports a previously unrecognized role for 5-HT signaling in pelvic ganglia ontogeny. We have previously demonstrated the robust expression of *Htr3a* in HuC/D+ neurons in the developing and mature pelvic ganglia [52]. However, our studies unexpectedly reveal that *Htr3a* is expressed not only in pelvic ganglia neurons but also in sacral NCPs prior to their differentiation that were isolated on the basis of *Sox10*-H2BVenus expression. Moreover, specific agonism of 5-HT3 via SR57227A resulted in a loss of neuronal fate acquisition in vitro and suggests that signaling through this receptor plays a role in lineage segregation. To our knowledge, this is the first study to implicate the 5-HT3 receptor in the specification of any cell type.

The 5-HT3 receptor is the only member of the 5-HTR family that functions as a ligand-gated ion channel [53] and is highly permeable to calcium. Given the extensive roles of calcium second-messenger signaling in neuronal development (reviewed in [54]), it is possible that neuronal specification may be influenced by calcium flux through this receptor. Calcium spike activity in the CNS has been shown to regulate the expression of genes driving neuronal specification, including *NeuroD*, while inhibiting the expression of *Hes1* and *Id2* that suppress neuronal fate [55,56]. Calcium flux further acts to modulate the specification of neuronal subtypes [57,58] and the acquisition of mature neuronal morphology [59,60] in the CNS. The potential role for 5-HT3-mediated calcium signaling to serve as a mechanism of neuronal specification and subsequent neuronal maturation is a ground-breaking principle that will require future analysis in vivo across multiple autonomic neuron populations to determine if this effect is restricted to sacral neural crest or more widely applicable to other NC populations in the peripheral nervous system.

Given the variety of pharmacological agents that are widely used as therapeutics for 5-HT modulation and the expression of additional 5-HTRs that we observed in developing pelvic ganglia neurons, there is broad potential for 5-HT receptor signaling to impact peripheral autonomic neuron development. To investigate this, we treated sacral NCPs with clozapine (mixed 5-HT2 receptor antagonist) and observed a modest effect on differentiation outcomes with an increase in purely neuronal colonies and concurrent reduction of colonies comprised of neurons and glia. Notably, we observed a pronounced effect of clozapine treatment on the multipotency of NC-derived progenitors with a significant reduction of colonies containing all three lineages of neurons, glial cells, and myofibroblasts. Although clozapine is known to antagonize multiple serotonin receptors [61], the 5-HT2A receptor is one candidate that may be the mediator of increased neuronal differentiation in our in vitro cultures. We readily detected *Htr2a* by RT-PCR in pelvic ganglia neurons captured on the basis of *Uchl1* transgene expression, and within the 5-HT2 receptor family, clozapine exhibits the highest binding affinity for 5-HT2A [62,63]. In contrast to our observations, a prior study using *Htr2a* knockout animals found that 5-HT2A is highly expressed in NC-derived enteric neurons, but the loss of this receptor did not alter the distribution of enteric neuron numbers or patterning of ganglia [64]. The difference between the pharmacological effects of clozapine that we observed may be due to action at other 5-HTRs or differences in 5-HT2A function between autonomic neuron populations that populate the bowel versus the sacral crest that populates the lower urinary tract. Regardless, our findings underline the importance of continued study of 5-HTR signaling in the development of NC-derived autonomic lineages in the lower urinary tract.

In addition to modulating NC differentiation potential, we found that over-stimulating the 5-HT3 receptor by the exposure of fetal pelvic ganglia to SR57227A led to a significant reduction in neurite arbor complexity. These findings are consistent with phenotypes reported in a prior study of 5-HT3 receptor mutant mice, *Htr3a^vs/vs^*, in which the 5-HT3 receptor was targeted by homologous recombination to produce a variant that is hyper-sensitive to serotonin binding and allows greater ion flux into the channel than wild-type receptors [65]. The bladder wall in *Htr3a^vs/vs^* mice exhibits deficits of neuronal processes, which is analogous to the reduced neurite arbor complexity we observed in pelvic ganglia cultures treated with 5-HT3 receptor agonist. The reduction of bladder wall innervation in *Htr3a^vs/vs^* was postulated to be the result of neurocytotoxicity, which is reasonable given the increased calcium signaling that would occur in these animals. Our data suggest that this effect occurs during development and that these developmental deficits of innervation persist into adulthood. *Htr3a^vs/vs^* animals also exhibit bladder smooth muscle hyperplasia. While reduced innervation in *Htr3a^vs/vs^* bladder could produce this muscle hyperplasia, it is intriguing that over-stimulation of the receptor results in increased bladder smooth muscle in vivo that is reminiscent of the increased myofibroblast differentiation we observed in SR57227A-treated cultures. Recent analysis from our group found that loss of the 5-HT3 receptor in *Htr3a* knockout mice results in increased innervation density in bladder smooth muscle in late fetal stages that is also evident in adult knockout mice [52]. Collectively, these findings suggest that signaling via the 5-HT3 receptor is crucial for the normal development of pelvic innervation and plays an important role in neuronal morphological growth and maturation in an activity-dependent manner. Our findings also suggest that roles for 5-HT3 previously documented in brain development, particularly in neuritic outgrowth and complexity [48,49,50], are conserved in the developing autonomic nervous system. Although our group and others have demonstrated roles for 5-HT3 signaling in neuronal process growth, the downstream molecular components mediating these effects remain unknown. Future efforts will be needed to determine how 5-HT3 activity regulates neuronal architecture during development.

Our studies add credence to the concept that the disruption of 5-HT signaling during development has the potential to cause enduring effects in the adult nervous system that predispose to long-lasting functional effects after birth. Accumulating evidence from studies of 5-HT signaling during brain development has shown that a variety of behavioral and molecular measures are impacted in adult animals when critical genes in 5-HT pathways are altered [66,67,68,69]. Other studies with variants of the serotonin transporter (SERT) have shown that 5-HT signaling is essential for normal neurogenesis within the enteric nervous system (ENS), including the intrinsic ganglia that mediate gastrointestinal motility [70]. In addition, the treatment of pregnant mice with pharmacologic compounds that alter 5-HT bioavailability can exert effects on aspects of both the CNS and ENS [70,71]. Our studies of sacral NC-derived progenitors and explanted pelvic ganglia presented here show that 5-HT signaling is also a key requirement for the normal development of pelvic ganglia neurons. Together with prior work that documented altered bladder innervation and function in *Htr3a* mutant mice (52), these studies collectively suggest that further study regarding the administration of compounds that alter 5-HT to pregnant women is justified.

As a whole, the experimental findings presented contribute to our understanding of signaling pathways that mediate sacral NC development and provide a valuable source of gene expression data that will fuel future studies of autonomic neurogenesis throughout the peripheral nervous system. Regional differences between axial levels of NC populations are well-established, but the sacral NC is relatively understudied compared to the cranial and truncal populations. The transcriptome dataset we have generated is a powerful resource for research groups interested in the identification of unique drivers of sacral NC development and comparing salient gene expression patterns between NC populations. Our discovery of the importance of serotonin receptor signaling, and specifically 5-HT3 as a mediator of neuronal specification in pelvic ganglia development, represents one of the myriad of applications of this transcriptome profiling study.

## 4. Materials and Methods

### 4.1. Mouse Husbandry

The Institutional Animal Care and Use Committee at Vanderbilt University Medical Center approved all animal procedures. Mouse strains utilized included Tg (*Sox10*-HIST2H2BE/Venus)^ASout^ (hereafter *Sox10*-H2BVenus, MGI: 3769269) and Tg (*Uchl1*-HIST2H2BE/mCherry)^FSout^ (hereafter *Uchl1*-mCherry, MGI: 5013570). Timed matings were set to obtain staged mouse fetuses, designating the morning of plug formation as 0.5 days post coitum (dpc). Transgenic mice were screened by tissue fluorescence and PCR as previously reported [22,24] using the oligonucleotide primers listed in (Table 2). Thermocycling parameters were: 94 °C, 5 min; 35 cycles of (94 °C, 30 s; 55 °C, 30 s, ramp 0.5 C/s to 72 °C; 72 °C, 30 s; ramp 0.5 °C/s to 94 °C); 72 °C, 10 min.

### 4.2. Isolation of mRNA from Fetal Mouse Lower Urinary Tract

Litters from timed pregnancies were harvested at 13.5, 14.5, or 15.5 days post coitus (dpc), counting the day of plug detection as 0.5 dpc. Embryos were screened for fluorescence in whole mount; then, pelvic ganglia were dissected and dissociated for flow sorting as described [22]. Pools of 4-18 intact fetal LUT or micro-dissected pelvic ganglia were combined to form single samples. At least three independent pools were collected for the harvest of RNA. Total RNA was isolated as previously described [22,24].

### 4.3. Immunohistochemistry (IHC)

Fetal cryo-sections were cut sagittally on a Leica cryostat at a thickness of 20 microns and mounted onto 3-aminopropyltriethoxysaline (3-APES) treated slides. After drying the slides at 37 °C for 30 min on a slide warmer, they were immersed in 1XPBS-0.3% Triton X-100 for 5 min to dissolve residual embedding medium. Then, slides were blocked for at least 30 min at room temperature in blocking solution (1XPBS-0.3% Triton X-100, 10% Bovine Serum Albumin (Sigma A2153, St. Louis, MO, USA), 5% Normal Donkey Serum (Jackson ImmunoResearch 017-000-121, West Grove, PA, USA), 0.45 μm sterile filtered). Slides were incubated in primary antibodies (Table 3) diluted in blocking solution overnight at 4 °C. The slides were rinsed thoroughly in sterile 1XPBS and incubated in secondary antibodies diluted in blocking solution for 1 h at room temperature (Table 4). After rinsing with sterile 1XPBS, the slides were immersed in 0.5 mM cupric sulfate in 50 mM ammonium acetate buffer to quench autofluorescence. Then, sterile water was applied to quench the cupric sulfate. The slides were mounted with AquaPolyMount (Polysciences, Inc., 18606, Warrington, PA, USA) and coverslipped.

### 4.4. Microarray Hybridizations and Data Analysis

RNA sample concentrations were determined by RiboGreen fluorescence (ThermoFisher, #R11490, Waltham, MA, USA) in comparison to a standard curve ranging from 1 to 50 ng/mL. For each sample, 3 ng total RNA was amplified by Nugen WT-PICO (Affymetrix, #3300, Santa Clara, CA, USA), processed on the Nugen Exon Module (Affymetrix, #2000, Santa Clara, CA, USA) and the FL-Ovation kit (Affymetrix, #4200, Santa Clara, CA, USA), followed by hybridization to mouse Gene 1.0 ST Affymetrix arrays. Raw data were processed with Affymetrix Gene Expression Console using robust Multi-Array Average (RMA) normalization [72]. Differential expression analysis was performed using the LIMMA Bioconductor package (Supplementary Table S1) [73]. A false discovery adjusted *p*-value of <0.05 was considered statistically significant. Heatmap and cluster analysis were performed using R package heatmap3 [74]. The top 100 up- and down-regulated genes were selected by ranking fold change relative to total embryo mRNA levels for each stage examined (13.5 dpc Supplementary Table S2; 14.5 dpc Supplementary Table S3; 15.5 dpc Supplementary Table S4).

### 4.5. Reverse Transcription (RT) PCR

cDNA was synthesized from purified RNA using the High Capacity cDNA Reverse Transcription kit (ABI, Life Technologies, #4368814, Carlsbad, CA, USA), with an input of 25 ng RNA and in accordance with the manufacturer’s instructions. All cDNA was stored in small aliquots at −80 °C to avoid repeated freezing and thawing. To minimize the consumption of cDNA, the TaqMan Pre-Amplification Master Mix kit (Life Technologies, #4384266, Carlsbad, CA, USA) was used to amplify cDNA templates for the specific detection of serotonin receptor genes (*Htrs*). Primers to detect serotonin receptor gene expression were carefully designed to bridge exons and avoid regions of high homology between *Htr* gene family members and detect the expression of all known splice variants (Supplementary Table S6). The pooled assay mix for the amplification contained reverse and forward primers for each murine *Htr* gene, along with primers for housekeeping gene *ActB* as a control. Pre-amplified cDNA was used for traditional PCR, using the same *Htr* primers pooled in the pre-amplification mix. Thermocycling conditions were the following: 94 °C 5 min; 34 cycles of (94 °C 30 s, 55 °C 30 s, 72 °C 30 s); final extension 72 °C 10 min. PCR products were visualized by non-denaturing polyacrylamide gel electrophoresis.

### 4.6. Assessment of Developmental Potential in Cultures of Sacral Neural Crest Progenitors

Fetal LUT tissues including bladder, pelvic ganglia, urethra, and genital tubercle were micro-dissected from 14.5 dpc *Sox10*-H2BVenus transgenic mice. Venus+ cells were isolated via FACS. Cells were plated and grown in culture using established conditions as previously described [26,28]. The cultures were treated with either drug vehicle control, clozapine (1 μM, Sigma Aldrich #C6305, St. Louis, MO, USA), or 5-HT3 receptor-specific agonist SR57227A (1 μM, Sigma Aldrich, #S1688, St. Louis, MO, USA). Cells were grown in self-renewal media for one week and then switched to differentiation media and cultured for another week. Drugs were replenished every three days over the differentiation media culture period. To determine the developmental potential of NCPs following exposure to these drugs, cultures were fixed and immunostained for differentiated cell type markers (peripherin for neurons, GFAP for glia, Smooth Muscle Actin (SMA) for myofibroblasts) and counter-stained with DAPI. Colony composition was determined by counting the total number of colonies per well that were labeled by each marker. Colonies were classified as being purely neuronal, glial, or myofibroblast (N, G, and M, respectively), or combinations of these lineages. Percentages of colony type were calculated by dividing the number of colonies of a particular subtype over the total number of colonies in the well. Then, these percentages were averaged across replicates.

### 4.7. Pelvic Ganglia Explant Cultures

Pelvic ganglia cultures were established as previously described [6]. Briefly, collagen (Millipore, 08-115, Burlington, MA, USA) was mixed with 5× DMEM (made from Gibco 12100 powdered media) to normal osmolality and adjusted to neutral pH (~6.9–7.4) using Phenol Red indicator dye. Collagen was diluted to 1 mg/mL final concentration in DMEM (Gibco, 31053, Waltham, MA, USA) containing 10% fetal bovine serum (Benchmark, 100-106) and penicillin, streptomycin, and amphotericin B (Gibco, 15240-96, Waltham, MA, USA). Pelvic ganglia from 13.5 dpc urogenital sinuses were visualized under fluorescence illumination based on expression of the *Sox10*-H2BVenus transgene [22,24] and micro-dissected into ice-cold PBS. Individual pelvic ganglia were transferred to single wells of a 6-well tissue culture plate coated with collagen gel and gently inserted into the gel surface using micro-forceps. Agarose beads (Sigma C1461, St. Louis, MO, USA) saturated with 100 ng/µL of NT-3 (Peprotech 450-03, Rocky Hill, NJ, USA) were positioned in the gel using a 27 g needle at a position 1000 µm from the explant center. A vehicle-treated bead was positioned at 1000 µm on the opposite side of each explant. For 5-HT3 agonist-treated collagen gels, SR57227A (Sigma, S1688, St. Louis, MO, USA) was reconstituted in sterile MilliQ water to 1000 µM and added to the media to achieve concentrations ranging from 10 to 50 µM. After four days of culture at 37 °C in 5% CO_2_, explants were fixed in neutral buffered formalin (NBF, Sigma, HT501128, St. Louis, MO, USA) with 0.1–0.5% TX-100 (Fisher BP151, Waltham, MA, USA) and processed for IHC detection by rinsing with wash buffer (PBS with 0.1% TX-100 with 0.5% Bovine Serum Albumin (BSA, Sigma, A2153, St. Louis, MO, USA)) and blocking in PBS with 0.1–0.5% TX-100 with 1% BSA and 5% Normal Donkey Serum (NDS, Jackson ImmunoResearch, 017-000-121, West Grove, PA, USA). Primary antibodies were diluted in blocking solution and applied to cultured explants overnight at 4 °C. Explants were rinsed in wash buffer and incubated in secondary antibody for 1–2 h at room temperature. Following staining, cultured explants were rinsed in wash buffer, post-fixed in 4% NBF with 0.1–0.5% TX-100, and imaged on a Leica DMI 6000B inverted fluorescent microscope.

### 4.8. ApopTag Assay for Apoptosis in Pelvic Ganglia Explant Cultures

After 4 days of culture, pelvic ganglia explants were fixed for 1 h at 4 °C with neutral buffered formalin (Sigma) and subsequently washed into 1 × PBS. Apoptotic cells were detected in intact explants with the ApopTag^®^ Red In Situ Apoptosis Detection Kit (EMD Millipore, S7165, Burlington, MA, USA) while still embedded in the collagen gel. Following this assay, the explants were subjected to immunochemistry with anti-Hu C/D antibody (a pan-neuronal marker, gift of V. Lennon, 1:10,000) overnight at 4 °C and a donkey anti-human Cy5 secondary at a 1:250 dilution for 1 h at room temperature. Explants were incubated for 5 min in 0.5 mg/mL DAPI and then transferred to glass slides and coverslipped using glycerin jelly (7.5% *w*/*v* glycerine, 55% *v*/*v* glycerol) and maintained at 4 °C in a dark, humidified chamber until imaging. Confocal microscopy was performed on a Zeiss Scanning Microscope LSM510 using a 633 nm laser for imaging Cy5 (649–745 band pass filter), 543 nm laser for imaging Cy3 (560–615 band pass filter), and a 488 nm laser for imaging Alexa 488 (505–550 band pass filter) to visualize the transgene expression and secondary antibody fluorophores. Images were captured with the Zeiss LSM Image Browser Software and then exported from the Image Browser software as.tiff files and assembled in Adobe Photoshop (2014 2.2 release, Adobe Systems Inc., San Jose, CA, USA).

### 4.9. Sholl Analysis

Neurite extension was measured in images of pelvic ganglia explants from each experimental condition (*n* = 12−14) that had been stained with PGP9.5. Individual images were converted to 8 bit/channel, indexed color tiff files in Adobe Photoshop (2014 2.2 release, Adobe Systems Inc., San Jose, CA, USA). Neurites in each image were traced semi-automatically using the NeuronJ plugin [75] for ImageJ (version 1.50e, National Institutes of Health, Washington, DC, USA) and saved as grayscale bitmap images. Arborization complexity was measured by Sholl analysis of neurite tracings using the Sholl ImageJ plugin [76]. For each image, the center of the ganglion was used as the center of analysis and the ending radius was defined by the most distal point of the neuritic arbor. A radius step size of 0 was used for continuous sampling of the entire arbor. The number of intersections between sampling radii and the arbor were assessed as a readout of arbor complexity.

### 4.10. Statistical Analysis

Colony composition among drug treatment groups (vehicle control, SR57227A, clozapine) was averaged for each treatment group. Colony percentage means were statistically compared via Welch’s *t*-test; a *p*-value of <0.05 was considered significant. For Sholl analysis, the number of radii intersecting the arbor of each sample per treatment group (vehicle control, 25 μM, and 50 μM SR57227A) were averaged. These averages were statistically compared via one-way ANOVA with Tukey’s Honest Significant Difference (HSD) post-hoc test to correct for multiple comparisons. The size of the enclosing radius of the arbor of each sample per treatment group was averaged and compared via one-way ANOVA with Tukey’s HSD post hoc test.

## Figures and Tables

**Table 1:** Expression levels of 5-HT receptor genes in developing pelvic ganglia.

		13.5 dpc NCP		14.5 dpc NCP		15.5 dpc NCP	
Gene Symbol	mRNA Accession Number	Fold Change	Adjusted *p*-Value	Fold Change	Adjusted *p*-Value	Fold Change	Adjusted *p*-Value
*Htr1a*	NM_008308	−0.16	0.5054	−0.48	0.1293	−0.07	0.8173
*Htr1d*	ENSMUST00000121571	0.21	0.4659	−0.24	0.6965	0.08	0.8384
*Htr1f*	NM_008310	0.73	0.0116	0.02	0.9902	0.56	0.1074
*Htr2a*	NM_172812	0.47	0.1181	−0.20	0.7986	−0.29	0.4381
*Htr2b*	NM_008311	−1.41	0.0006	−0.97	0.0475	−1.44	0.0011
*Htr2c*	ENSMUST00000096299	1.39	2.08 × 10^−5^	−0.51	0.2132	−0.06	0.8892
*Htr3a*	NM_013561	1.21	0.0008	2.33	2.18 × 10^−7^	2.55	4.16 × 10^−9^
*Htr3b*	NM_020274	0.61	0.2917	1.79	0.0112	2.15	0.0010
*Htr4*	NM_008313	0.81	0.0047	0.08	0.9422	0.48	0.1593
*Htr5a*	NM_008314	0.43	0.1824	−0.07	0.9622	0.29	0.4627
*Htr5b*	NM_010483	1.17	1.72 × 10^−5^	0.05	0.9626	0.24	0.4069
*Htr6*	NM_021358	0.52	0.0398	−0.25	0.6064	0.44	0.1555
*Htr7*	NM_008315	1.46	3.77 × 10^−5^	−0.05	0.9748	0.35	0.3744

Fold change of serotonin receptor mRNA relative to total fetus mRNA levels for samples at ages 13.5, 14.5, and 15.5 dpc. Adjusted *p*-values were corrected for multiple statistical comparisons. Note that *Htr3a* is significantly enriched in differentiating pelvic ganglia throughout the developmental time window of autonomic neurogenesis and differentiation.

**Table 2:** Oligonucleotide primers used for genotyping mouse lines in this study.

Genotype	5′ to 3′ Sequence	Expected Product Size
*Sox10* BAC vector arm insert junction	Sox10 BAC Sp6 Forward: GTTTTTTGCGATCTGCCGTTTC Sox10 BAC Sp6 Reverse: GGCACTTTCATGTTATCTGAGG	227 bp
	Sox10 BAC T7 Forward: TCGAGCTTGACATTGTAGGAC Sox10 BAC T7 Reverse: AAGAGCAAGCCTTGGAACTG	202 bp
*Sox10*-H2BVenus	Internal Forward: CTGGTCGAGCTCGACGGCGACGTA Internal Reverse: AGTCGCGGCCGCTTTACTTG	580 bp
*Uchl1* BAC vector arm insert junction	Uchl1 BAC Sp6 Forward: GCCGTCGACATTTAGGTG Uchl1 BAC Sp6 Reverse: CCTACCCTTCGTCTTCTTTTG	200 bp
	Uchl1 BAC T7 Forward: GTGTTGCTTTCTTTGAGTGG Uchl1 BAC T7 Reverse: TACTCAGGATGCTGAAACAGG	257 bp
*Uchl1*-H2B	Internal Forward: GTACTAAGGCCGTCACCAAG Internal Reverse: GTACATGAACTGAGGGGACAG	263 bp

**Table 3:** Primary antibodies used in immunohistochemistry.

Antigen	Host	Vendor, Catalog Number	Dilution	RRID
PGP9.5	Rabbit, polyclonal	AbD Serotec, #7863-0504	1:4000	AB_2210505
Peripherin	Rabbit, polyclonal	Millipore, #AB1530	1:1000	AB_90725
FITC-conjugated FITC	Mouse, monoclonal	Sigma Aldrich, #F3777	1:800	AB_476977
Cy3-conjugated GFAP	Mouse, monoclonal	Sigma Aldrich, #C9205	1:800	AB_476889
HuC/D	Human	Gift of Dr. Vanda Lennon, Mayo Clinic	1:10,000	N/A
DAPI	N/A	Invitrogen, #D1306	1:50,000	N/A

**Table 4:** Secondary antibodies used in immunohistochemistry.

Antigen	Vendor, Catalog Number	Dilution	RRID
Donkey anti-Rabbit AlexaFluor 647	Jackson ImmunoResearch, #711-605-152	1:250	AB_2492288
Donkey anti-Human AlexaFluor 647	Jackson ImmunoResearch #709-605-149	1:200	AB_2340578

## Data Availability

The datasets generated in this study are publicly available through the Gene Expression Omnibus accession viewer repository (GSE108943, GSM778845-GSM778854).

## Supplementary Materials

The following are available online at https://www.mdpi.com/article/10.3390/ijms22136838/s1, Supplementary Table S1: Output from LIMMA Bioconductor package; Supplementary Table S2: Top 100 genes E13; Supplementary Table S3: Top 100 genes E14; Supplementary Table S4: Top 100 genes E15; Supplementary Table S5: Colony Counts; Supplementary Table S6: HTR gene specific primers.
