# Supplementary material for: 5-HT3 Signaling Alters Development of Sacral Neural Crest Derivatives That Innervate the Lower Urinary Tract
Source: Int J Mol Sci. 2021 Jun 25;22(13):6838. doi: 10.3390/ijms22136838 (PMC8269166; doi:10.3390/ijms22136838)
Supplement: Supplementary file 1 [file ijms-22-06838-s001.zip › Ritter_Buehler_Supplementary Table S5_Colony Counts_v3.pdf]

Supplementary Table S5. Effect of Serotonergic Drugs on Sacral Neural Crest Developmental Potential.

Treatment	Number of Total Colonies	Percentage of Cell Type						
		N	N+G	G	N+M	M	G+M	N+G+M
Untreated (n=45)	21.4 ± 5.4	45.2 ± 1.69	12.8 ± 1.38	1.1 ± 0.41	32.2 ± 2.04	0.3 ± 0.19	0.0 ± 0.0	8.3 ± 1.20
SR57227A (n=42)	22.1 ± 3.5	31.3 ± 1.33***	8.9 ± 1.18*	1.0 ± 0.37	51.2 ± 1.78***	1.0 ± 0.31	0.1 ± 0.11	6.4 ± 0.80
Clozapine (n=33)	24.4 ± 5.5	50.9 ± 1.72*	8.8 ± 1.28*	0.5 ± 0.24	36.6 ± 2.23	0.1 ± 0.09	0.0 ± 0.0	3.1 ± 0.81**

Percentages of colony types (N = neuronal, G = glial, M = myofibroblasts) relative to total number of colonies per well. Values shown are average ± S.E.M. SR57227A and Clozapine treated were compared to Untreated colonies [*Significant ($p<0.05$), **Very Significant ($p<0.01$), ***Highly Significant ($p<0.001$)].
