# Supplementary material for: 5-HT3 Signaling Alters Development of Sacral Neural Crest Derivatives That Innervate the Lower Urinary Tract
Source: Int J Mol Sci. 2021 Jun 25;22(13):6838. doi: 10.3390/ijms22136838 (PMC8269166; doi:10.3390/ijms22136838)
Supplement: Supplementary file 1 [file ijms-22-06838-s001.zip › Ritter_Buehler_Supplementary Table S6_Htr Primers_v1.pdf]

Supplementary Table S6. Primers used to detect serotonin receptor expression in reverse-transcription PCR experiments.

Gene Name	Forward Primer (5' to 3')	Reverse Primer (5' to 3')	Product Size (bp)
Htr1a	CTTTTCCTAGAACACGCAGCTT	TTGGGATTTCGAATAGAGAGGA	194
Htr1b	GTCTCCTGTGTACGTGAACCAA	GGCATCCTTACAGATAGGCATC	184
Htr1d	CGAGAAAGGAAAGCCACTAAGA	TGACGGGGTTAATGAGAGAGTT	181
Htr1f	GAGGAACTGTTAAACCGAATGC	TCACCAGGACAGCTACAAGAAA	190
Htr2a	GCTACAGGATGATTCGAAGGTC	TGGAAGAGCTTTTCTGATGACA	246
Htr2b	GCAGGTACATCACCTGCAATTA	GAGGAGGATGATTGATGAGGAC	209
Htr2c	ATTGGTTTGGCAGTTCGATATT	TGCAGTTTCTTCTCCATGCTTA	204
Htr3a	CTTAGGTCCTGCATCCTGTTTC	TTGACGCCCTGATAAGTAAGGT	181
Htr3b	GGGGTTACAACATGTAGGGAAA	GCTTGCTTGTCACTGCATACTC	199
Htr4	CACACAGTAGCAGCTCAAAACC	GCTTACATTTGGGTCCTCTGAC	178
Htr5a	GCTTTCAAGGTCTTCTTCTCCA	GGAAGAGTGCCCAGACATAAAC	250
Htr5b	CCAAGTCCCTGAATAATGGAAG	CCAGTAGAGTCACCACAAGCAC	193
Htr6	TGGCTGGGATACTGTAATAGCA	CCCTGAAGCTGAGTCTGAATCT	237
Htr7	AAACACAAGTTCTCAGGCTTCC	AGGTAGTGGCTGCTTTCTGTTC	172

All *Htr* primers were intentionally designed to bridge exons and avoid regions of high homology between *Htr* gene family members. For genes with known splice variants, primers were designed to allow amplification of any splice variant that might be present.
